# Noble Precision: Advancements in Silver (Ag) Radiation for X-ray Crystallography

**DOI:** 10.1063/4.0001051

**Published:** 2025-10-27

**Authors:** Michael Ruf, Ashley Schmidt, Tobias Stürzer

**Affiliations:** 1 Bruker AXS, LLC, Madison, WI, USA; 2 Bruker AXS SE, Karlsuhe, BW, Germany

## Abstract

Harness the precision of Silver (Ag) radiation, a noble choice for modern X-ray crystallography. With its shorter wavelength of 0.56 Å, Silver radiation offers significant advantages over traditional Molybdenum (Mo) radiation, establishing it as the preferred option for small molecule crystallography. Recent technological advancements have greatly enhanced the performance of Ag X-ray systems, providing sources that exceed the intensity of conventional Mo X-ray microfocus sources and modern detectors that exhibit high quantum efficiency for Ag radiation. Today’s Ag diffraction systems offer unparalleled access to crystallography applications, significantly improving the performance and precision of crystallography laboratories. This presentation will explore the transformative impact of Ag radiation on home-lab crystallography, highlighting its benefits and the cutting-edge technologies driving its adoption. Examples and comparisons to Mo radiation will be presented to underscore the superior capabilities of Ag radiation.

